# A Commentary on “Risk Factors for Renal Cell Cancer in a Japanese Population”

**DOI:** 10.4137/cmo.s2926

**Published:** 2009-08-14

**Authors:** Esther Uña Cidon

**Affiliations:** Medical Oncology Service. Clinical Universitary Hospital, Valladolid (Spain).

**Keywords:** renal cell cancer, risk factor, Japan

## Abstract

The well-written and researched article reported in *Clinical Medicine: Oncology* by Dr. Washio and Dr. Mori entitled “Risk factors for renal cell cancer in a Japanese population”^1^ makes evident the differences in incidence and mortality rates from renal cell carcinoma (RCC) between different populations and highlights the relevance of carrying out epidemiological studies, investigating additional risk factors which may explain the differences.

The incidence of RCC, which is the most common type of kidney cancer, has been increasing worldwide, mainly in western countries, in the last three decades at a rate between 2% and 4% per year.^2^–^4^ Accumulating evidence has confirmed this data.

In the world 208,000 new cases are diagnosed and 102,000 die from this disease each year. More than one half have been diagnosed in the United States.^3^

This incidence rate of RCC varies internationally more than 10 fold, with the rates of RCC in Asian countries being lower than those of countries in North America or Europe.^3^ This fact suggests a strong role for exogenous risk factors, in addition to possible roles of geographic differences in genetic susceptibility and diagnostic variability.

However, which factors that have influenced this increase have not been easy to determine.

On the one hand, the relatively recent improvements in diagnostic techniques and the extensive use of the computed tomography for studying other diseases, may have contributed to this rising incidence with improved earlier diagnosis. Most tumors diagnosed in this way are asymptomatic and therefore incidental findings. These findings are supported by the study published by Bretheau et al^5^ which confirms that the incidence of these asymptomatic RCC have increased from 14% in eighties to 48% in nineties.

However on the other hand, I would like to highlight that both incidence of late stage RCC detected by symptoms and mortality secondary to it, have also been increasing, implying that risk factors are playing an important roll in this upward trend as well.^6^

Therefore at this point we must ask ourselves who’s at risk, and why?

There are many epidemiological studies carried out in different countries around the world that seek answers to this question. Although an essential precondition for any epidemiological study is to identify the relevant physical factors or environmental agents involved in a determined disease, our lack of basic knowledge does not allow us to design an epidemiological study which is powerful enough to resolve a small risk,^7^ even if such a risk truly exists. This is the reason why different studies have identified multiple risk factors for RCC, many of them with a weak causative association, so the question remains unanswered.

Although multiple high-risk groups for RCC have been truly identified (obesity, smoking and hypertension), with a marked consistency, taken together, these risk factors account only for 49% of cases, so a large portion of RCC still has an unknown and unexplained etiology.^6^ This fact implies that the search for additional risk factors must continue. In this context observational epidemiological studies remain a very important way of identifying causal associations between risk factors and disease. These studies should reliably identify powerful causal associations.^8^–^10^

Although we know there is racial disparity and geographical differences in this disease, we have to obtain almost all this data predominantly from non-Asian patients (Caucasian in Europe, and Caucasian and African American in the United States). This is not the ideal as the incidence of RCC is higher in Japanese Americans than in native Japanese suggesting an implication of environmental factors such as life-style as Dr. Washio and Dr. Mori highlight in their article.

Conversely RCC incidence has been increasing in this population too. Thus I consider as they have done, that it is very relevant to study risk factors for RCC between native Japanese which clearly will become important to obtain strong conclusions and to prevent it. As Dr. Washio and Dr. Mori have suggested, I too consider it is valuable to study, among other factors, the implications of a Japanese diet against a western diet, although I am aware of this is not easy because the difficulties of obtaining meaningful dietary histories in a homogeneous population in which the social environment could also play a roll.

Since this malignancy is rare, all epidemiological studies have a common limitation related to small numbers of cases in certain risk factor categories. Because of this it is difficult to reach statistical significance,^7^ so reporting an association between a factor and a disease concluding causation is not always possible. Environmental epidemiology is very difficult to do. Although many risks and associations have turned up in the laboratory, they are not well-established in human epidemiological studies. The risk factors most widely recognized by the scientific community are limited to obesity, hypertension and smoking.

Establishing this link is not as easy as I first thought. Although obesity is a well established risk factor for RCC in Western countries^11^ with a relative risk of 3.3 (95% confidence interval 1.2–8.7) in women and 2.3 (95% confidence interval 1.2–4.5) in men. In this study obesity shows a meaningful association with RCC but a marginal association with RCC death when the researchers excluded those cases with a medical history of diabetes mellitus (after adjustment for age and sex, relative risk was 1.69 with confidence interval between 0.87–3.30). The increasing prevalence of obesity may partly explain the increasing incidence of RCC. In fact, the proportion of RCC attributable to overweight and obesity is estimated to be more than 40% in the United States and more than 30% in Europe. I would also like to point out that in the study by Bretheau et al^5^ they observed that the rate of incidental RCC was higher in groups of patients who underwent computed tomography for studying cardiovascular diseases or biliary diseases, and they concluded that the prognosis was better in these groups on finding these incidental tumors, therefore the rate of death secondary to RCC was lower. We know that this group of diseases is related among other factors with obesity, so this fact could have some implication in these results, therefore making the interpretation of epidemiological data more difficult.

Diabetes mellitus has been associated with an increased risk in several cancers, however its relationship as an increased risk in regard to RCC is controversial and indicates a moderate, if any, increased risk. Only scanty information is available on the RCC risk in patients with diabetes. In the study by Zuchetto et al^12^ in an Italian population, when they compared patients with and without diabetes, those with a diagnosis of diabetes mellitus had a relative risk of 1.25 (95% confidence interval 0.91–1.73). The risks of RCC in relation with diabetes were similar across the strata of sex, body mass index and smoking.

Dr. Washio and Dr. Mori have determined that diabetes mellitus is not a significant risk factor in the development of RCC. Research has shown an increased risk of dying from RCC and a higher risk of death from RCC after excluding obese subjects without diabetes. Globally we can conclude that we need further prospective studies to evaluate the link between obesity and diabetes in the etiology of RCC because we don’t know if the association is true or confounded by obesity.

We have to deal with a similar problem with hypertension and its treatment as Dr. Washio and Dr. Mori explain. Association between diuretic treatment and RCC has been showed in a few prospective studies. The problem is that they have been used as treatment for hypertension, so it is difficult to separate their effects from the effects of hypertension.^6^

In this Japanese population there is an association between hypertension and RCC incidence and also death with a relative risk of 4.27 and 1.98 respectively (95% confidence interval 2.07–8.79 and 1.06–3.70 respectively).

Smoking has been related to an increased RCC risk for both sexes. For men a significant increase in risk was observed among past smokers (relative risk 1.51 (1.08–2.11)) and current smokers (relative risk 2.30 (1.55–3.41)) and a significant dose-response trend with pack-years of smoking has been noted. Men who had smoked more than 20 pack-years had 2.3 fold increased risk compared with non-smokers. Among women although the trend was not as strong as for men, there was also a dose-response relation with pack-years detected.^6^

Despite these results which are based on western studies, in this Japanese population smokers showed a marginally increased risk of dying from RCC.

These controversial results highlight the small statistical power of such studies which is a common limitation of epidemiological studies carried out in this disease because of small number of cases and deaths secondary to it.

I believe the main point to stress is diet, mainly because of the differences between western and Asian countries. These authors have concluded that a Japanese diet with starchy roots (such as taro, sweet potatoes and potatoes) was associated with a decreased risk of dying from RCC. However to the contrary there is no meaningful association with fruits and vegetables that have been advocated as protective factors in western countries.

Drinking of black tea, which is considered as a surrogate for westernized diet increases the risk of RCC deaths after adjustment of the other factors.

On reflection of these controversial results I have made the following interpretations regarding the above mentioned risk factors.

There has been much discussion about the difficulties in reliably determining moderate effects, mainly when we are talking about behavioural risk factors that involve an element of choice such as diet or drinks or life-style habits.

Epidemiological studies could be useful tools in identifying possible health hazards providing sufficient reliability in differentiating between exposed and non-exposed subjects to these risk factors.

We must bear in mind that as Bretheau et al^5^ and others have published, the early diagnosis of this disease significantly impacts on overall survival so they conclude that the patients with incidental tumors had a better prognosis than those with symptomatic tumors because of lower tumoral size and local stage. The most important conclusion we can obtain from their article is that the earlier the diagnosis of RCC, the better the prognosis for these patients.

It is in this context where it would be relevant to be able to stratify populations based on risk factors for RCC because this would allow establishment of strategies to prevent this disease which could have a major public health impact. The most important limitations to achieve this aim are the difficulties in considering not only the baseline exposures but to consider changes during follow-up too. However we must not forget the exposure to many chemical agents or diet because these substances might not be effectively metabolized.^13^

The global differences mainly in results related with dietary habits need to be further explored in metabolic and laboratory investigations that may lead to clinical intervention studies and public health guidelines or recommendations. Epidemiology functions best when operating in a clinical environment with close access to laboratory studies.

Despite all these common limitations, Dr. Washio and Dr. Mori carried out a proper epidemiological study which allows us to better identify the risk factors of this rare malignancy in a different population as was performed with Japanese people, whose behavioral habits, mainly dietary habits, are different from our own.
